# Desiring support on a winding road with challenging intersections: Social and professional support for sexual minority forced migrant men

**DOI:** 10.1111/jan.16256

**Published:** 2024-05-29

**Authors:** Tommy Carlsson, Rummage Isaac, Ronah Ainembabazi, Anna Eldebo, Sumera Yasin, Maria Gottvall

**Affiliations:** ^1^ The Department of Women's and Children's Health Uppsala University Uppsala Sweden; ^2^ The Department of Health Sciences The Swedish Red Cross University Huddinge Sweden

**Keywords:** forced migrants, lesbian, gay, bisexual, transgender, and queer (LGBTQ+), psychosocial health, public health nursing, sexual and gender minorities

## Abstract

**Aim:**

To explore experiences of social and health professional support among sexual minority forced migrant men.

**Design:**

Exploratory qualitative study.

**Methods:**

Individual semi‐structured interviews were conducted in 2023 with 15 participants recruited through convenience, purposive and snowball sampling. Interviews were audio recorded, transcribed and analysed with systematic text condensation in a collaborative process between researchers and experts by lived experience.

**Results:**

The first category was ‘desiring support along a road with challenging intersections’. Participants encountered a harsh reality and dangers in the host country. They sought social connections and communicated with others whilst in a social labyrinth within a new and reserved society. Although social support was desired and highly appreciated, the process involved a spectrum of both belonging and exclusion. The second category was ‘navigating uncharted waters when seeking affirming health services’. A range of barriers to health services were encountered in a complex health system. Participants emphasized the importance of safe and affirming spaces that accommodate the vulnerability of disclosure.

**Conclusion:**

Ensuring respectful and affirming support for sexual minority forced migrants is essential. Barriers in accessing health services need to be addressed, including informing about rights and ensuring safety.

**Implication for the Professional and Patient Care:**

Nurses and other health professionals can consider social support as a potentially valuable resource for health promotion. However, there is a need for more research investigating its mental health effects.

**Impact:**

The intersectional disadvantages and discrimination encountered by sexual minority forced migrants call attention to the need for further advancements in inclusion health and affirming care.

**Reporting Method:**

This study adhered to the Consolidated Criteria for Reporting Qualitative Research.

**Patient or Public Contribution:**

Three sexual minority forced migrants were members of the research team. They were involved in the data collection, analysis and reporting in close collaboration with researchers.

## 1 INTRODUCTION

Worldwide, persons identifying as lesbian, gay, bisexual, queer, or having other sexual orientations beyond societal norms are subjected to discrimination, pathologization, criminalization and persecution (Mendos et al., 2020). In countries where individuals of sexual diversity are oppressed, they experience significant violence and abuse (Alessi et al., 2021). As a response to the global mistreatment of individuals with diverse sexual orientations, leading international organizations emphasize the importance of ending violence and discrimination against these populations (ILO et al., 2015). Living under dangerous circumstances and not being able to live authentically leaves many affected persons with no other alternative than to seek asylum in other countries. Fear of persecution due to sexual orientation is a valid reason for asylum in many countries (UN, 2019). Sexual orientation can be viewed as an enduring physical, romantic, emotional and/or spiritual attraction to another person. Henceforth, we will use the term sexual minorities when referring to people whose sexual orientation is not self‐identified as exclusively heterosexual.

## 2 BACKGROUND

Research has shown that the mental health of sexual minority individuals is overall poorer when compared to the general population (Pitoňák, 2017). This includes mental health issues such as depression, anxiety, suicidal ideation, self‐harm and substance abuse (King et al., 2008). Compared with heterosexual counterparts, sexual minority cisgender men report elevated levels of depression, anxiety and suicide attempts (Plöderl & Tremblay, 2015). Studies suggest that sexual minority forced migrant men experience health‐related burdens and unmet health needs during resettlement (Gottvall et al., 2023; Yarwood et al., 2022). Moreover, studies indicate that these men are exposed to violence and abuse (Gottvall et al., 2023; Yarwood et al., 2022) while encountering challenges trying to establish themselves in society and trying to find safe housing (Waite et al., 2019).

Forced migration involves a loss of social relationships, contributing to isolation and mental burdens (Ekoh et al., 2023). Among sexual minority forced migrants, loneliness is a prominent concern associated with psychological distress (Fox et al., 2020). Social support is considered a determinant of health among forced migrants (Rashki Kemmak et al., 2021) and can act as a protective factor in the development of mental health burdens (Schlechter et al., 2021). Since long, a range of positive effects of social support has been acknowledged in the literature (Cohen & Wills, 1985). Herein, we address social support within a larger system of societal structures, networks and interpersonal relationships (House et al., 1988). Sexual minority forced migrant men express a need for social support when resettling in the host country (Gottvall et al., 2023). Some form strong bonds with peers during resettlement, while distancing themselves from family members and other migrants (Karimi, 2020). However, research investigating the experiences and dimensions of social support exchanged between sexual minority forced migrant men is scarce.

Host countries have a responsibility to ensure access to adequate health services for those seeking asylum. Nurses and midwives have an ethical and professional obligation to provide respectful and adequate support to sexual minorities as well as forced migrants (Dorsen & Van Devanter, 2016; ICN, 2021; Wilson et al., 2022). Regrettably, forced migrants encounter a range of barriers when seeking professional support (Lebano et al., 2020; WHO, 2021). Insufficient knowledge about the available services and stigma are contributors to low utilization of health services among forced migrants in general (Satinsky et al., 2019). Studies about health service utilization among sexual minority men highlight general as well as specific barriers related to heterosexism and homophobia (Gaspar et al., 2021). Despite the call for inclusive and affirming clinical settings (Stokes & Lecuyer, 2023), there is a need to achieve higher clinical awareness on how to support sexual minority individuals (Baptiste‐Roberts et al., 2017). Little has been reported about the experiences of accessing and utilizing health services among sexual minority forced migrants. Limited research highlights that professional support offers important tools for psychological adaptation and relief (Gottvall et al., 2023). In one recent study within the Canadian context, sexual minority forced migrants described challenges in accessing safe and affirming care (Haghiri‐Vijeh, 2022). The lack of research calls attention to the need for additional studies about how health services are accessed and how they can be improved.

## 3 THE STUDY

### 3.1 Aim

The aim of this study was to explore the experiences of social support and professional support from health services among sexual minority forced migrant men.

## 4 METHODS

### 4.1 Design

An exploratory qualitative study was conducted through a collaboration between two researchers and three sexual minority forced migrants (referred to as experts by lived experience). In line with recommendations for public contribution, the researchers hired experts by lived experience as research assistants and engaged with them in close collaboration through weekly workshops and in‐depth discussions (Salsberg et al., 2015). The experts by lived experience were recruited with purposive methods through the established networks of the researchers and were employed as research assistants throughout the study. This study is reported according to the Consolidated Criteria for Reporting Qualitative Research (Tong et al., 2007).

### 4.2 Theoretical framework

The theoretical underpinnings supporting this study relate to intersectionality (Crenshaw, 1989) and minority stress (Meyer, 2003). Intersectionality acknowledges that power, privilege and discrimination are shaped by the unique social identity of each person. From an intersectional perspective, societal disadvantages based on multiple grounds involve a heightened risk of psychological distress (Vargas et al., 2020). According to the minority stress model, health‐related burdens among sexual and gender minority individuals stem from societal structures imposing stigma and marginalization (Flentje et al., 2020; Meyer, 2003; Pitoňák, 2017). These theories aided in the rationale and were utilized to view the findings through theoretical lenses. As an inductive approach was utilized, no theory was used as a template for coding into pre‐determined categories. The inductive approach was chosen to generate findings based on the data, and is a suitable method when conducting research with the goal of reaching novel insights about sparsely studied topics (Patton, 2015).

### 4.3 Study setting and recruitment

The study was conducted in Sweden, which acknowledges fear of persecution due to sexual orientation as a valid reason for asylum. While there are no published reliable statistics on asylum claims based on sexual minority status in Sweden, one report from the United Kingdom suggests that numbers vary by year and can reach up towards 7% of total number of applications (Home Office, 2023). Undocumented migrants and asylum seekers have the right to receive healthcare that cannot be deferred (The National Board of Health and Welfare, 2023). Sweden has implemented laws and policies promoting sexual minority equality and non‐discrimination, including recognition of same‐sex marriage and adopting children (Ministry of Employment, 2022). Various non‐governmental organizations provide support for sexual minority forced migrants.

Participants were recruited between April and June 2023 through convenience sampling by online advertisements (published via the social media channels for the university and at the university website), purposive sampling via the networks of the experts by lived experience, and snowball sampling through the participants who had been interviewed. The researchers had no relationship with participants prior to participation. An interest to participate was expressed via a digital application tool or by calling a member of the research team. A time and place for the interview was scheduled according to the preferences of the participant. Participants received a gift card of SEK 500 after completing the interview.

### 4.4 Inclusion criteria

Participants met inclusion criteria if they had experience of coming to Sweden as a forced migrant, self‐identified as a sexual minority cisgender man and were ≥18 years of age.

### 4.5 Data collection

The first and last authors conducted semi‐structured individual interviews. In line with the preferences of the participants, all interviews were conducted in English at one instance, without an interpreter being present. An interview guide developed a priori by the research team was utilized during the interviews (Table 1). Interviews were initiated with the question “please tell me a little about yourself.” When needed, the researchers asked clarifying and follow‐up questions. During interviews, interviewers took field notes, which were summarized for participants at the end of the interview to present them with an opportunity to clarify and correct any misinterpreted information. The interview setting was determined together with the participants. Fourteen were conducted face‐to‐face (seven at a university campus, seven at a hotel) and one was conducted digitally via a video conferencing tool. A research assistant with lived experience as a sexual minority forced migrant attended a proportion of the interviews. Before the interviews began, the interviewers introduced themselves to the participants and provided information about the study. All interviews were recorded on a digital audio file, transcribed and de‐identified. The length of the interviews ranged between 34 and 127 min. No transcripts were returned to participants following the conclusion of the interview.

**TABLE 1:** Interview guide with questions about social and professional support.

Topic	Main question	Follow‐up questions
Psychosocial health and well‐being	How has your situation been since you came to Sweden?	How has your psychological health been?
		How has your physical health been?
		What has been difficult for you since you arrived?
		What larger challenges have you encountered?
		How would you describe your social situation from when you first arrived in Sweden up until today?
Psychosocial support	What kind of support have you experienced during your time in Sweden?	What have you done to make yourself feel good or to improve your health and well‐being?
		Have you received the support you needed?
		What kind of support do you feel has been missing?
		Do you have any examples when you didn't receive the support you needed from health services?
		Have you ever felt discriminated against when in contact with health services, and if so, in what ways?
Competence in health services	How have you experienced the contact with and the treatment by health services/health professionals?	What do you think is important for health professionals to consider when they support sexual minority forced migrants?
		Do you think health professionals have enough knowledge about sexual minorities and forced migrants?
Areas for improvement and interventions	How do you think the support for sexual minority forced migrants can be improved in Sweden?	What could be improved in the contact and treatment with health services/health professionals?
		What kind of support would have been good for you to receive after arriving in Sweden?
		How do you feel health services can be improved to better meet the support needs of sexual minority forced migrants?

### 4.6 Data analysis

Transcribed materials were analysed in a collaborative process according to the procedures of systematic text condensation (Malterud, 2012). Researchers and experts by lived experience engaged in close collaboration throughout the steps of the analysis, involving individual work followed by joint discussions. First, transcribed interviews were read, and preliminary themes were identified. Second, meaning units were sorted into code groups derived from the preliminary themes. Third, meaning units were sorted into subgroups illustrating the content within each code group. Fourth, a condensate acting as an artificial quote based on the meaning units was produced for each of the subgroups. Fifth, illustrative quotes were identified and synthesized statements were written. In the final step, the analysts scrutinized the synthesized statements and produced category headings, acting as expressive and creative statements illustrating the most important content of each code group. The analysis was an iterative process, in which the analysts moved back and forth between the different steps and the transcribed interviews. The researchers and experts by lived experience were involved in all steps of the analysis. Additionally, two psychologists contributed as clinical consultants in the first and last steps of the analysis. While we did not strive for saturation, a tendency towards thematic saturation was noted as more interviews were conducted and analysed. No participant checking of final findings was performed. Throughout the analytic steps, analysts continuously searched the material for negative cases that would contradict or nuance the findings. Microsoft Word and Microsoft Excel were used to structure the meaning units into code groups.

### 4.7 Ethical considerations

This study was approved by the Swedish Ethics Review Authority (approval number: 2022‐01483‐01). All participants were provided oral and written information about the study and gave written informed consent to participate. All participants were informed about the study by a researcher, and all potential participants had the right to decline without needing to state a reason why. All interviews were de‐identified and provided a study‐specific code number in place of names. Only those analysing the data had access to de‐identified transcripts, and only researchers in charge of the study had access to the raw data. All research team members worked under confidentiality.

### 4.8 Rigour and reflexivity

The research team consists of a group of individuals with varied expertise and perspectives, which were capitalized during the analysis. Throughout the study, the research team engaged in mutual discussions to challenge preconceptions and reach findings they all felt represented the data. The principal investigator took memos throughout the process. The main researchers do not have lived experience of being forced migrants. Both are Swedish‐born researchers and nurse–midwives with experience of qualitative data collection, self‐identifying as a woman and as a genderqueer person respectively. To challenge their preconceptions and view the data from diverse perspectives, they actively collaborated with experts by lived experience with the intention to co‐produce qualitative findings that are enriched and nuanced (Flicker & Nixon, 2015).

## 5 RESULTS

### 5.1 Characteristics of participants

Table 2 presents sample characteristics. Participants had a range of migration statuses, including undocumented migrants, asylum seekers and having been granted asylum. Based on the information provided, participants migrated to Sweden because of their sexual minority status (either as one of several reasons, or as the main reason). Several were in the process of seeking asylum based on persecution experiences related to sexual minority.

**TABLE 2:** Characteristics of the included participants (*n* = 15).

Characteristics	*n* (%)
Age	
20–29	8
30–39	6
40–49	1
Level of education	
College/university	9
High school	5
Elementary school	1
Years residing in Sweden	
≤1	9
2–3	3
4–5	3
Sexual orientation	
Gay/homosexual	14
Bisexual	1
Region of origin	
Africa	10
Asia	4
Not disclosed	1

### 5.2 Qualitative findings

Following their arrival in the host country, sexual minority forced migrant men embarked on a journey of desiring company on a winding road with challenging intersections. They sought social connections and communicated with others while navigating a social labyrinth within a new and reserved society containing hard realities. While social support was desired and highly appreciated, social relations involved feelings of both belonging and exclusion. When needing health services, participants navigated uncharted waters, which required courage and determination. A range of barriers to access adequate health services were encountered, and the importance of safe and affirming spaces that accommodate the vulnerability of disclosure was emphasized.

#### 5.2.1 Desiring social support along a road with challenging intersections

##### Harsh realities of intersectional vulnerability and dangers

Participants encountered a harsh societal reality based on intersectional disadvantages when resettling in Sweden. Initially, participants were left in a state of confusion where to turn for safe housing. An unstable and unsafe housing situation continued over longer periods, as participants stayed in housing they could not afford and were thrown out with short notice. The housing situation caused significant mental pressure. Challenges in attaining and sustaining employment were also described. A lack of formal documents and living as undocumented hindered legal work and made them further susceptible to exploitation. Faced with intersectional discrimination, participants were turned down work or were fired because of their appearance and their sexuality.

[After arrival in Sweden] I just stayed like five days in the station and tried to eat something, before I went to immigration. [Interviewer: Okay, so that must have been a little scary.] Exactly yeah, scary afraid, because I see police, maybe they will arrest me or something, and it's cold. […] I don't feel safe [where I stay now] because that person is just like an alcoholic person, he stresses people. (Participant A)

In many aspects, Sweden was considered a friendly and welcoming country for sexual minorities. However, participants had encountered intersectional social exclusion, discrimination and judgmental behaviours within the general population. Participants encountered racism within the sexual minority community, while simultaneously encountering homophobia within the community of other forced migrants. Several felt unsafe when placed in asylum accommodations with migrants who displayed violent and homophobic behaviours. Fearing how migrants would react if they would find out about their sexuality, participants needed to conceal their sexuality and felt as if they still were under the same oppression as in their country of origin.

There are people from my country living there with me [in the asylum accommodation]. And you know, it's the same like [country of origin]. I felt the same, because they were with me the whole time, twenty‐four seven. […] Imagine as gay person living with these straight people who have this conservative mind, it's very hard. So, that's why like still you're hiding also yourself. So, that's why it's very stressful. (Participant E)

Participants talked about their experiences of being exposed to violence and abuse following their arrival in the host country. Some people in society took advantage of them as a newly arrived forced migrant. Participants were exposed to violence and abuse from disrespectful and abusive partners who exploited them. Partners had tried to control them and took advantage of them sexually. These relationships were described as toxic, and when participants ended these relationships, some partners had started spreading rumours and blackmailing them.

I met a guy from [country] and he was around my age. That was the worst experience. He was… I don't know. First, I met him in the summer, two weeks, and I was not… I did not have sex before. And then he forced me to sex. (Participant F)

##### Navigating a social labyrinth within a new and reserved society

Participants missed their social connections in their country of origin, including family and friends they had left behind. Several expressed feelings of loneliness while resettling in Sweden. They tried to establish social contacts in Sweden via face‐to‐face and online settings, including social activities, nightlife and dating apps. However, they encountered difficulties establishing a social network because of the reserved behaviours within the general population and because of their societal position as newly arrived sexual minority migrants. Not knowing the language and lacking money or formal identification needed to access social scenes further hindered networking.

I don't find people. I am trying. But it's not like, it's not working that much. I'm trying to date people, but some dating apps needs money. (Participant A)

Several felt relief when arriving in Sweden, looking forward to living openly as a sexual minority migrant. However, challenges to live out these expectations had been encountered as several expressed a need to continue concealing their sexuality in different settings. Moreover, participants felt unfamiliar in being open about their sexuality, particularly among those who represented the context and norms they fled from. This resulted in taking a reserved position in social settings and leading a double life, in which they felt a need to act and disclose information differently depending on social contexts.

Every time you try to open up about it, you know, you're not sure how everyone is going to react to it from back home up to here. Because some of the guys we meet in the LGBTQ groups and [non‐governmental organization] and some of them actually haven't been able to open up and like speak out. Because, even when I'm in there, I'm just quiet or I just try to make the jokes. But I don't speak too much because I don't know how. (Participant B)

##### Appreciating social support but sometimes encountering exclusion

Participants engaged in emotional, instrumental and informational social support with friends, partners and peers. Interacting with people who accepted and acknowledged them was validating and eye‐opening, helping them stay positive and hopeful. Peer support involved an opportunity to receive empathy and kindness without prejudice or judgement in a safe setting where they could express themselves comfortably. Participants also valued the instrumental social support received in the form of temporary housing, food, clothes, free gym cards and financial assistance. They received needed information from social contacts about societal norms, legal rights and boundaries, lawyers, public transportation, health services and employment opportunities.

[Interviewer: How do you feel about friends that you met at the non‐governmental organization?] Yeah, it is nice, amazing. Because first time I go there looking for my type. It's a lot of people in this meeting, and meeting, talking. That time, I really felt at home and [like] I found my new house, a new family. […] That time, I really felt relaxed. (Participant C)

Various peer support activities arranged through non‐governmental organizations and churches were attended by participants, including sports, games, sessions to practise language, library visits, movies and dancing. Through these activities, participants established deep and meaningful friendships with peers. Participants also appreciated activities to meet Swedish citizens, who helped them learn the language and understand society. Mental health support groups arranged by non‐governmental organizations were highly valued and desired, alleviating psychological distress and sleeping difficulties. Additional and longer mental health sessions together with peers and health professionals were desired.

In [non‐governmental organization], one psychologist came and explained some things in one day, but it should be more than two hours or something. He made it simple, and you couldn't ask him a lot of questions. (Participant A)

Interacting with peers also involved negative experiences of polarization and exclusion. Some participants did not feel welcomed at non‐governmental organizations, which they experienced as focused on certain subgroups within the umbrella of sexual and gender minorities. Participants had experienced differences in the resources from non‐governmental organizations which were provided to different migrants. Moreover, some had experienced a lack of boundaries and respect among peers, who they felt asked inquisitive and uncomfortable questions. At times, peers had stated negative comments about participants, resulting in them stopping attending further activities. Others continued attending sessions despite negative experiences.

There's an LGBTQ group, community called [organization]. […] I used to go to the meetings but now I don't because… you know, it's too many people asking too many questions, and sometimes you don't want to say everything out. So, I stopped going. That's why I couldn't even like get some of the profits everyone there is getting. (Participant D)

#### 5.2.2 Navigating uncharted waters when seeking affirming health services

##### Encountering barriers in pursuit of adequate health services

Participants described several barriers that hindered access to health services, leading to frustration and a lack of needed care and support. Being unfamiliar with the complex structure of the health system led to confusion, further compounded by the fact that little information about the health system had been provided by authorities. Not knowing the language resulted in difficulties finding information and booking appointments, requiring participants to rely on the assistance of social contacts that would help them. Queues for health appointments were experienced as long, and once they received an appointment they needed, it required money they did not have.

[Psychologist] is very amazing, but unfortunately, I think I can't go more. Because I don't have money, because I'm an asylum seeker. (Participant G)

When living as undocumented migrants, participants hesitated to seek care that they needed because they thought that they were ineligible. Later, they had understood that this information was incorrect, as undocumented migrants are indeed eligible for healthcare that cannot be deferred in Sweden. They also had heard that health professionals would report to authorities if they encountered an undocumented migrant, which made them fear potential consequences of seeking support. This misinformation and fear resulted in not seeking medical care despite needing it.

I stayed for one year without papers. I was undocumented. […] So if I want to get any medical help, I had to for it pay by myself, [Migration Agency] told me. And for that reason, I didn't go to any medical or whatever, for a long period of time. But you know what, I googled and I went to the [organization]. I went there and I asked them. They told me, you have rights and they told me that I have the right for any medical assist. […] But I didn't know, I was in pain. (Participant E)

##### The importance of compassionate and affirming communication in health services

Respectful and welcoming communication and structures were considered essential within health services. It took significant courage and safety for participants to disclose information about their background and sexual minority identity. Participants felt unsure how health professionals would react if they would disclose information about their sexuality and emphasized structures that promote comfortability in disclosing such information. To feel comfortable, participants needed health professionals to be friendly, open‐minded, understanding, affirming and empathetic. For some, it had been easier to open up to health professionals who they already knew or who were women.

I think a doctor or a nurse, they need to be friendly. Someone who is not friendly, someone who is just talking is not good. You need to be friendly, behave good, the nurse or doctor need to learn more from me. (Participant C)

However, some negative experiences of interaction with health professionals were described. These interactions made participants feel ashamed and hesitant to seek further care. Some health professionals had been in a hurry during their appointments and were inattentive to their needs. Additionally, some health professionals had not addressed topics related to sexual health and were experienced as having insufficient knowledge about the health needs of sexual minorities. Participants also mentioned that some health professionals had shown racist behaviours.

They have to learn themselves. That's a problem, the ignorance you know. They have to learn, they have to be more, they should have more empathy, you know. They should know. That's a problem. [Interviewer: They lack knowledge?] Exactly. They don't know how to approach the LGBTQ. (Participant E)

Participants addressed language as a primary barrier to communicating about their health and needs. However, varied views regarding the use of interpreter services were described. Some preferred to have an interpreter present to ensure they could describe their condition and understand health professionals correctly. Others felt hesitant because of fears about how the interpreters would react and handle confidentiality issues if their sexuality would be brought up. The risk of using an interpreter that would gossip and act judgmentally led to some using friends as interpreters. Alternatively, some decided to speak English with health professionals despite the communication difficulties. Participants regarded telephonic interpretation as an appropriate and comfortable option for sexual minority forced migrants, due to the possibility of being anonymous and avoid negative consequences of disclosure.

The interpreters are also there from [country of origin]. And I always felt, you know… I'm not open yet. I'm still in closet. And the problem from [region of origin] also, from… they're not only judgmental, but the gossip is like [snaps his fingers]. […] You always have the fear you know. So, that's why I like… it's very hard to bring someone who is from your background, from [country of origin], and to translate for you. I don't speak English well, but for me it doesn't matter. It's better for me to speak broken English than to expose myself. (Participant E)

## 6 DISCUSSION

### 6.1 Principal results

This study aimed to explore experiences of social and professional support among sexual minority forced migrant cisgender men. The study presents novel findings about the dimensions of vulnerability and support as sexual minority forced migrants resettle in contexts similar to Sweden. Echoing studies in other contexts (Alessi et al., 2021), participants were placed in a vulnerable position in society. The findings are in line with the minority stress model (Meyer, 2003) and the theory on intersectionality (Hill Collins & Bilge, 2016). In sexual minority populations, societal marginalization is associated with loneliness (Elmer et al., 2022). Limited previous research indicates that sexual minority forced migrants are at risk of experiencing impactful loneliness (Fox et al., 2020) and intersectional vulnerability (Gottvall et al., 2023). Our study brings insights into the intersectional layers of loneliness and adds to the Temporal Intersectional Minority Stress Model (TIMS) (Rivas‐Koehl et al., 2023). In line with the proximal stressors presented in the TIMS, our participants described intersectional disadvantages and risks, including interpersonal violence and abuse. These findings call attention to the importance of taking into consideration the unique circumstances that shape vulnerability and resilience. Nurses and other health professionals need to ensure that health services support sexual minority forced migrants who live in poor and unsafe conditions. Screening for exposure to intimate partner violence is a valuable tool to identify individuals in need of support and protection (Rollè et al., 2018), which is further echoed by our findings.

As illustrated by our findings and the TIMS, social support has the potential to buffer the negative mental health effects of intersectional disadvantages and proximal stressors leading to minority stress (Cohen & Wills, 1985; Rivas‐Koehl et al., 2023). Peer support is highlighted as one of six key principles for trauma‐informed approach (SAMHSA, 2014). In line with our findings, previous research suggests that social support is desired and valued by sexual minority forced migrants (Alessi, 2016; Gottvall et al., 2023). Similarly, the importance of social support as a resource to ameliorate the effects of stressors is emphasized in the minority stress model (Meyer, 2003; Rivas‐Koehl et al., 2023). Indeed, social support seems to be a promising intervention to alleviate health burdens for forced migrants in general (Mahon, 2022) and for hardly reached populations (Sokol & Fisher, 2016). However, there is a scarcity of research investigating its effects among sexual minority forced migrants. Interestingly, some participants experienced exclusionary practices and polarization within the community of sexual minority forced migrants. The double‐edge nature of social relationships is well documented in the literature, as social settings can involve unsupportive behaviours leading to negative impacts on health and well‐being (Taylor, 2011). At an individual level, social support can provide emotional relief and is a potential intervention for clinical utilization. However, all aspects of support need to be taken into account when referring migrants to sources of peer support, including arranging follow‐up discussions with clients.

Previous research illustrates the importance of support for sexual minority asylum seekers, who experience high levels of chronic health burdens and unmet health needs (Gottlieb et al., 2020). Host countries have a responsibility to ensure access to professional support for asylum seekers. Limited research highlights that professional support offers important tools for psychological adaptation and relief (Gottvall et al., 2023), which was echoed in our study. However, forced migrants encounter a range of barriers when seeking professional support following their arrival in the host country (Lebano et al., 2020; WHO, 2021). Moreover, sexual minority men experience specific barriers to adequate health services (Gaspar et al., 2021). Insufficient knowledge about available support and stigma are known contributors to low utilization of health services (Satinsky et al., 2019). Further barriers articulated by our participants included unfamiliarity with the health system, not knowing their rights to health services, and not knowing the language. The specific barriers encountered by undocumented migrants call attention to the need to apply a temporal perspective on minority stress experienced among sexual minority forced migrants (Rivas‐Koehl et al., 2023). At a systemic level, society needs to increase access to health services through enhanced information dissemination to forced migrants. Nurses and other health professionals have an important role to ensure that forced migrants are provided with support by breaking down the barriers hindering access.

The Ethical Code by the International Council of Nurses states that nurses are obliged to provide respectful and unrestricted care for patients and clients based on human rights (ICN, 2021). However, little has been reported about the experiences of accessing and utilizing health services among sexual minority forced migrants. In our study and in the Canadian context, forced migrants of sexual minorities describe challenges in accessing safe and affirming care (Haghiri‐Vijeh, 2022). In line with these findings, ensuring a safe and welcoming setting is highlighted as a key principle in trauma‐informed clinical approaches (SAMHSA, 2014) and when establishing clinical practices for sexual minority individuals (Mukerjee et al., 2021). Ensuring a welcoming and inclusive communication and environment is essential, as individuals of sexual minorities in general fear poor treatment if disclosing intimate information (Brooks et al., 2018; Mukerjee et al., 2021). Strategies to achieve a safe and inclusive clinical setting involve establishing a partnership with the client, applying a person‐centred approach, and engaging in purposeful self‐reflection (Mukerjee et al., 2021). According to our findings, insufficient knowledge among health professionals and fears related to confidentiality when using interpreters are important clinical considerations. The utilization of interpreters when supporting sexual minority migrants is a complex topic in need of further attention in research. Nurses and other health professionals should ensure that forced migrants are assessed about their preferences regarding interpreters to promote comfortability. These findings highly align with TIMS (Rivas‐Koehl et al., 2023), emphasizing a tailored approach taking into account the multidimensional unique stressors faced by disadvantaged sexual minority populations.

### 6.2 Strengths and limitations of the work

In this qualitative study, participants were recruited through a combination of convenience, snowball and purposive sampling to achieve a varied sample. Considering the limited size of this population and the fact that we utilized snowball sampling, it is probable that participants had social connectedness. We do not know the exact number of participants recruited through snowball sampling, and readers should note that some may stem from similar social networks. We included participants from countries in Africa and Asia, which we believe represents common countries that sexual minority forced migrants flee from. Nevertheless, we cannot disregard the possibility that interviews with participants from other countries or regions and older individuals would generate additional findings. As this study focused on the experiences among cisgender men, this study needs to be interpreted together with additional research including additional subgroups self‐identifying as sexual and gender minority forced migrants.

Readers need to consider the context of this study when interpreting the transferability. The processes and access to support may vary between host countries, as rights to healthcare and the organization of non‐governmental organizations differ. Based in Sweden, the transferability of this study may be limited in relation to other settings that differ from the study context. Participants were interviewed by two researchers who are nurse–midwives experienced in qualitative studies. While we approached the data collection by using a semi‐structured guide and open‐ended questions, it is possible that the backgrounds of the interviewers influenced the data.

To address biases, the analytic process involved a collaborative process between researchers and experts by lived experience. Co‐analysing qualitative data together with representatives of the target population have the potential to enhance rigour and can result in democratized findings enriched through diverse perspectives (Flicker & Nixon, 2015). We argue that the analytic approach capitalizing on diverse perspectives enhanced the trustworthiness.

### 6.3 Recommendations for further research

We encourage future research developing and evaluating peer support interventions for sexual minority forced migrants. Future research should address methods to reduce the barriers to health services encountered by sexual minority forced migrants. There is a need for studies investigating models for affirming clinical support and how to achieve an appropriate utilization of interpretation services in clinical settings. We encourage more research investigating methods about communication and competence among health professionals. The findings highlight the need for exploration of the experiences among other subgroups within the umbrella of sexual and gender minorities, for example, cisgender women, transgender and non‐binary individuals.

### 6.4 Implications for policy and practice

The findings emphasize culturally sensitive and affirming communication in health services, which could be achieved through training about the unique and intersectional challenges faced by sexual minority migrants. Health professionals need to ensure accessible health services that address health burdens faced by sexual minority forced migrants. Minority stress, intersectional discrimination, risk of exposure to violence and loneliness can further inform clinical practice that promotes safety, acceptance and validation. Health professionals should assess the need for social support via non‐governmental organizations that can complement support from health services. However, an awareness of the potential risk of exclusionary practices within communities is needed. The use of interpreters needs to be assessed on an individual basis, with consideration of risks related to confidentiality and client comfortability.

## 7 CONCLUSION

Sexual minority forced migrant men experience intersectional disadvantages and are at risk of developing mental health burdens. Nurses and other health professionals need to consider the risk of unsafe housing situations impacting the health and opportunities for support among sexual minority forced migrants. Screening for intimate partner violence is a valuable and important tool to identify individuals in need of support and protection. While social relationships have the potential to provide highly valued support between sexual minority forced migrants, there is a need for more research investigating the potential benefits and negative effects of peer support. Barriers in accessing health services need to be addressed, including informing undocumented migrants and asylum seekers about their rights for support. Nurses and other health professionals must ensure that they communicate in a respectful and affirming manner when supporting sexual minority forced migrants, including appropriate utilization of interpreters in line with the client's preferences.

## AUTHOR CONTRIBUTIONS

All authors made substantial contributions to conception and design, or acquisition of data or analysis and interpretation of data; involved in drafting the manuscript or revising it critically for important intellectual content; gave final approval of the version to be published. Each author should have participated sufficiently in the work to take public responsibility for appropriate portions of the content; and agreed to be accountable for all aspects of the work in ensuring that questions related to the accuracy or integrity of any part of the work are appropriately investigated and resolved.

## FUNDING INFORMATION

This research was funded by the Swedish Research Council for Health, Working Life and Welfare (Forte) [grant number: GD‐2021/0028].

## CONFLICT OF INTEREST STATEMENT

The authors declare that they have no conflicts of interest.

### PEER REVIEW

The peer review history for this article is available at https://www.webofscience.com/api/gateway/wos/peer‐review/10.1111/jan.16256.

## Data Availability

The data are not publicly available due to privacy and ethical restrictions.
